# Efficient mutagenesis and genotyping of maize inbreds using biolistics, multiplex CRISPR/Cas9 editing, and Indel-Selective PCR

**DOI:** 10.1186/s13007-025-01365-w

**Published:** 2025-03-25

**Authors:** Maruti Nandan Rai, Brian Rhodes, Stephen Jinga, Praveena Kanchupati, Edward Ross, Shawn R. Carlson, Stephen P. Moose

**Affiliations:** 1 Department of Crop Sciences, College of Agricultural, Consumer and Environmental Sciences (ACES), University of Illinois at Urbana-Champaign, Champaign, IL 61801 USA; 2 DOE Center for Advanced Bioenergy and Bioproducts Innovation, University of Illinois, Champaign, IL 61801 USA

**Keywords:** Gene editing, High throughput genotyping, Indel-Selective PCR (IS-PCR)

## Abstract

CRISPR/Cas9 based genome editing has advanced our understanding of a myriad of important biological phenomena. Important challenges to multiplex genome editing in maize include assembly of large complex DNA constructs, few genotypes with efficient transformation systems, and costly/labor-intensive genotyping methods. Here we present an approach for multiplex CRISPR/Cas9 genome editing system that delivers a single compact DNA construct *via* biolistics to Type I embryogenic calli, followed by a novel efficient genotyping assay to identify desirable editing outcomes. We first demonstrate the creation of heritable mutations at multiple target sites within the same gene. Next, we successfully created individual and stacked mutations for multiple members of a gene family. Genome sequencing found off-target mutations are rare. Multiplex genome editing was achieved for both the highly transformable inbred line H99 and Illinois Low Protein1 (ILP1), a genotype where transformation has not previously been reported. In addition to screening transformation events for deletion alleles by PCR, we also designed PCR assays that selectively amplify deletion or insertion of a single nucleotide, the most common outcome from DNA repair of CRISPR/Cas9 breaks by non-homologous end-joining. The Indel-Selective PCR (IS-PCR) method enabled rapid tracking of multiple edited alleles in progeny populations. The ‘end to end’ pipeline presented here for multiplexed CRISPR/Cas9 mutagenesis can be applied to accelerate maize functional genomics in a broader diversity of genetic backgrounds.

**Supplementary Information:**

The online version contains supplementary material available at 10.1186/s13007-025-01365-w.

## Introduction

Maize is the global leader for grain production, with more than one billion tons harvested annually [12]. Evaluation of natural variation in industrial breeding programs has led to dramatic improvements in maize yields worldwide [45]. The recent burst of genome data and functional genomics resources for maize has uncovered multiple novel targets for further improvement. CRISPR/Cas9 technology is utilized to generate mutations in targeted genomic regions in many plant species including maize [43]. However, multiplexing CRISPR/Cas9 genome editing presents a number of technical challenges, including assembly of constructs with multiple DNA elements, a limited number of RNAP III promoters typically used to drive sgRNA expression, inefficient transformation protocols, and subsequent genotyping to identify and follow inheritance of edited mutations.

One solution to reduce size and complexity of editing constructs is to express multiple sgRNA scaffolds as one polycistronic mRNA separated by processing elements, each driven by a single RNA polymerase II promoter. In addition to simplifying construct assembly using Type IIS restriction-mediated cloning approaches such as GoldenGate, RNA polymerase II promoters allow for targeted expression in certain tissues or at precise developmental times [13, 40] and are thus more versatile than RNA polymerase III promoters. In one example of this approach, Qi et al., [35] utilized the existing tRNA processing pathway in plants and introduced flanking tRNA processing sites within a transcript containing multiple guide RNAs driven by a single RNA polymerase II promoter. Two additional multiplexing strategies that have been shown to function in plants are self-cleaving ribozymes [19] and the Csy4 ribonuclease that mediates bacterial processing of CRISPR arrays [18, 31]. A compact vector system has been developed for assembly and Csy4 processing of multiple gRNAs expressed from the constitutively active Cestrum yellow leaf curling virus promoter [7], with successful demonstration of multiplexed editing in crops such as soybean [30], rapeseed [47], and tomato [37].

Another bottleneck in CRISPR/Cas9 mutagenesis of maize is the requirement to deliver editing components to regenerable tissue cultures, which can be reliably induced from only a few genotypes. The most efficient maize genotype for *Agrobacterium*-mediated transformation, Hi-II, is a hybrid, potentially complicating phenotypic analysis of progeny. First reported by Lowe et al., [29, 28] and with recent improvements [46], use of the morphogenic regulators BABYBOOM and WUSCHEL dramatically expands the genotypes available for transformation. However, these methods still require careful optimization of expression, with some of the key elements being governed by intellectual property that may limit access by academic groups.

Even when multiplex genome editing is successful, efficient identification of individual alleles among the initial population of mutations or their inheritance among progeny can be challenging. Although genotyping ‘drop-out’ mutations (deletion of > 10 bp) can be done easily by simple PCR and gel electrophoresis, such assays do not reveal single nucleotide insertion/deletion (indel) mutations that are the most common editing outcome from Cas9 in plants [3]. Both DNA sequencing and in vitro RNP CRISPR/Cas9 digestion assays of amplicons spanning gRNA target(s) can readily identify single-base edits, but are not cost-effective for large-scale screening or introgression of CRISPR/Cas9 derived alleles into additional genetic backgrounds of interest.

The current work presents an approach aimed to address each of the above challenges for multiplex genome editing in maize. Multiple gRNAs were cloned into the compact Csy4 vector system developed by Čermák et al. [7]. Single DNA fragments containing each of Cas9, gRNA scaffolds, and selectable marker gene were delivered via biolistics to Type I embryogenic calli, which can be established from a much broader range of maize genotypes [11]. We demonstrate successful multiplexed genome editing in two different maize inbred lines by creating heritable mutations at multiple target sites within the same gene, as well as multiple members of a multigene family. Lastly, we developed a novel and efficient PCR-based assay to selectively amplify single-base indel mutations, and apply Indel-Selective PCR to genotype populations for desirable combinations of edited alleles.

## Results

### DNA design for multiplex editing of the maize *Lemon White1* gene

Multiplex editing is achieved with either multiple constructs expressing individual components (nuclease, gRNAs, selectable marker) or single constructs that express all components. Constructs expressing individual components are easier to build and offer more flexibility in design, but the frequency of codelivery decreases with the number of constructs. Single constructs are desired but can present difficulties in the cloning and propagation of large (often greater than 15-kb) inserts with repeated elements, such as RNAP III promoters or gRNA scaffolds. The vectors designed by Čermák et al. [7] address many of these issues by expression of multiple gRNAs as a polycistronic mRNA, driven by the compact CmYLCV promoter [7]. Expression of an N-terminal fusion of the Csy4 processing protein to a monocot codon-optimized Cas9, separated by the P2A self-cleaving peptide, is controlled by the strong constitutive *ZmUbi1* promoter. The editing components are coupled to a selectable marker gene for recovery of transgenic events. GoldenGate entry sites facilitate both direct cloning of multiple synthesized gRNA scaffolds into a common backbone, or modular assembly of different promoters and editing components.

**Fig. 1:**
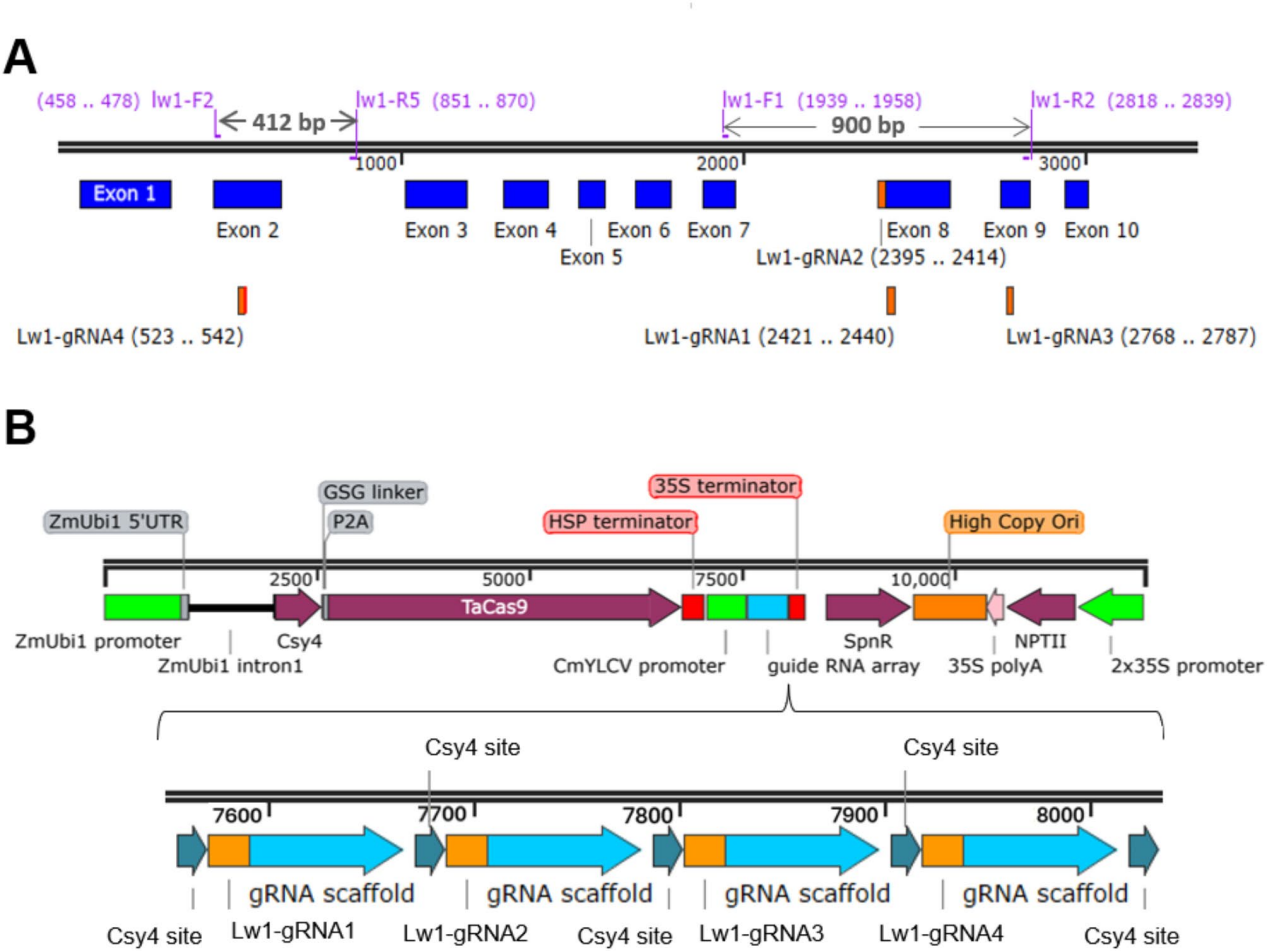
DNA design for multiplex editing of the maize *Lemon White1* gene **(A)***Lemon White 1* (*LW1*) gene model depicting exons and the position of designed guide RNAs. **(B)** Schematic design of editing DNA construct delivered by biolistics for multiplex *LW1* gene editing

To assess functionality of the Csy4 multiplexed editing system in maize, we selected the *Lemon White 1 (LW1*, Zm00001eb056240*)* gene for a proof-of-concept experiment because of prior success in generating easily visualized yellow/albino leaf phenotypes by CRISPR/Cas mutagenesis [14, 41]. Four guide RNAs were designed to target *LW1* exons using the B73 reference genome assembly (Supplementary Table 1). The LW1-gRNA1 generated edits previously [14] and was included as a positive control (Fig. 1A), along with three gRNAs targeting either upstream (LW1-gRNA2, LW1-gRNA4) or downstream (LW1-gRNA3) exons (Fig. 1A). All four LW1-gRNAs were assembled into the pMOD_B2103 destination vector, which was then combined with plasmids containing *ZmUbi1*:Csy4-P2A-TaCas9 and the CaMV35S:*nptII* selectable marker (Fig. 1B). TaCas9 was codon-optimized for expression in wheat (*Triticum aestivum* L.) and other monocots. The editing function of this vector was initially tested with in vitro Cas9 RNP cleavage assays, (supplemental Fig. 1) where each of the three newly designed gRNAs produced expected cleavage products when tested individually, as well as in combination.

### Multiplex editing of the *LW1* gene in the H99 and ILP1 maize inbred lines

The majority of genome edits created to date in maize have been generated in only a few inbred genotypes, such as B104 [1, 23] that produces fast-growing embryogenic callus tolerant of co-cultivation with *Agrobacterium.* We sought to develop genome editing for the broader diversity of inbreds capable of forming slower-growing Type I callus, which have been successfully transformed following DNA delivery by biolistics [16, 44]. We chose to edit the H99 inbred because of its historically high transformation efficiency (3% in Brettschneider et al., [5] nearly 30% in Shiva Prakash et al., [38]. To further demonstrate the versatility of the approach, we also chose to generate mutations in the inbred line Illinois Low Protein1 (ILP1). ILP1 is derived from the Illinois Long-Term Selection Experiment for grain protein concentration [32], exhibits high nitrogen utilization efficiency [42] and harbors novel genetic variants for improving maize yield, seed composition, and nitrogen use efficiency [49].

**Fig. 2:**
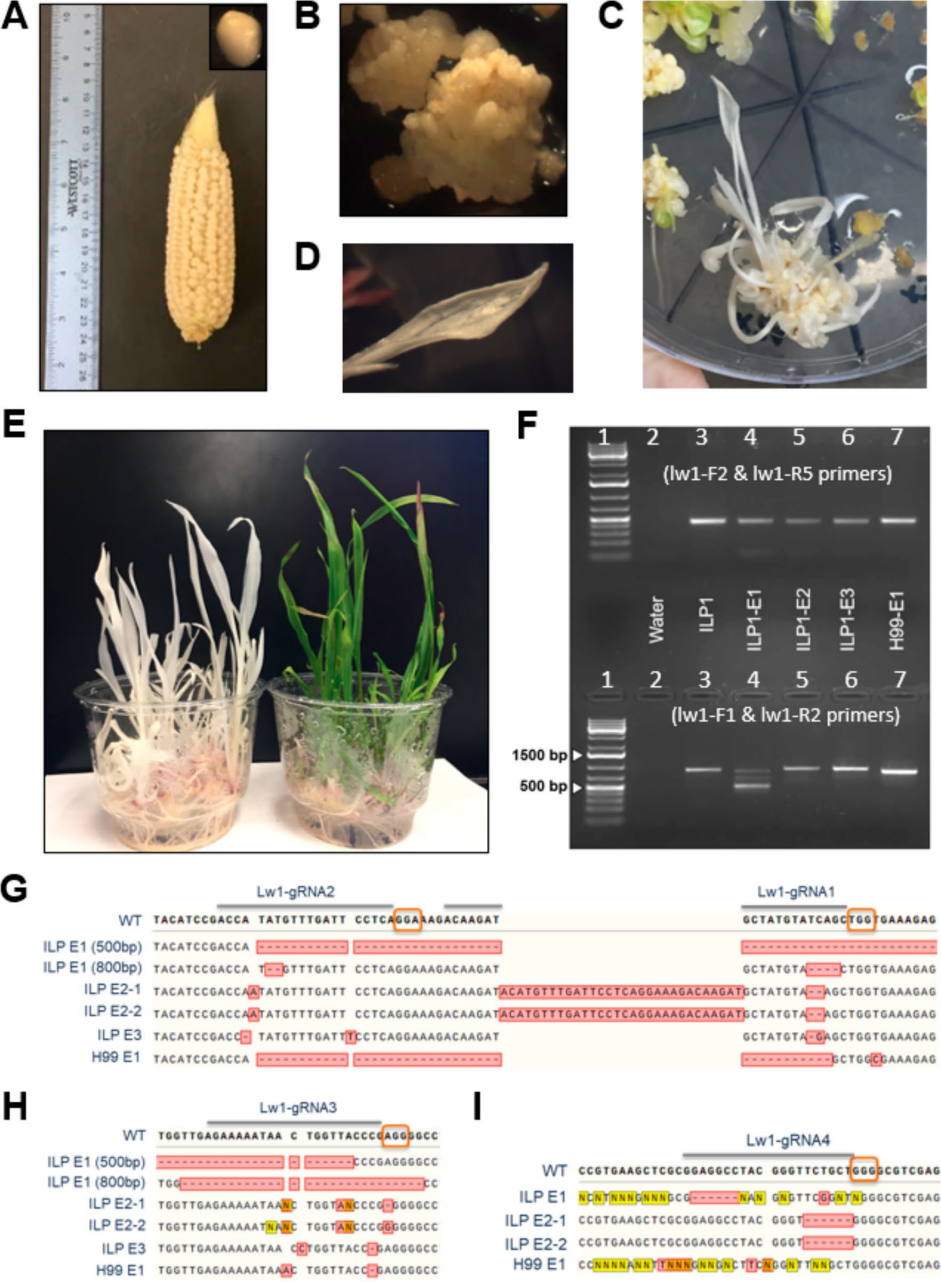
Multiplex genome editing of *LW1* in H99 and ILP1 inbred lines. **(A)** Selfed ears of ILP1 harvested 12–14 days after pollination, with excised immature embryo in inset. **(B)** Putative transgenic calli on selective medium. **(C)** CRISPR/Cas9-events on selective regeneration medium. **(D**,** E)** Putative transgenic shoots on selective regeneration medium after 2–3 weeks post-transfer to a growth chamber with 16 h/8 h light/dark photoperiod. **(F)** Sequence confirmation of genome edits in *LW1*. The top half of the gel shows amplification of the 5’ end of the gene spanning the *LW1*-gRNA4 target site, bottom half shows amplicons spanning target sites for *LW1*-gRNA1, *LW1*-gRNA2 and *LW1*-gRNA3 at 3’ end of gene. Primer locations are indicated in Fig. 1A. Lane 3 (ILP1) is the non-edited control plant producing a band of 900 bp. Lanes 4 (ILP-E1), 5 (ILP-E2), 6 (ILP-E3) and 7 (H99-E1) are the edited lines. Lane 1 is the NEB 1 kb + ladder. **(G**,** H**,** I)** Sanger sequencing results of PCR products shown in panel F (guide RNA targets are depicted with gray line above WT sequence, PAM sequences are depicted in orange boxes; red boxes with dashes and letters represent deletions and insertion, respectively

The single 12-kbp gel-purified DNA fragment for *Lw1* editing (Fig. 1B) was used to transform immature embryos of the H99 and ILP1 genotypes by biolistic transformation. Following methods optimized by Shiva Prakash et al., [38] transgenic events harboring *lw1* mutations were generated for both H99 and ILP1 (Fig. 2). As anticipated, embryogenic calli induced from ILP1 exhibited the morphogenic features of Type I embryogenic calli (Fig. 2B). Approximately 12 weeks post-bombardment, putative transgenic shoots appeared on selective regeneration medium (Fig. 2C). Table 1 shows transformation efficiency of H99 in this experiment was comparable to prior reports (4.5%), and a slightly higher frequency of 7.2% was observed for ILP1. Importantly, more than 20 events were produced for both genotypes. The transformation frequencies observed here are also similar to those recently achieved via biolistics transformation of Type I callus from inbred B104 [36]. Transgenic plantlets were observed to display either partial or fully albino leaves (Fig. 2) indicative of *lw1* mutations.

**Table 1:** Transformation efficiency (regenerated events per bombarded embryo) of independent experiments with immature embryos of H99 and ILP1

Experiment	H99			ILP1		
	Bombarded embryos	Independent events that regenerated	Transformation efficiency (%)	Bombarded embryos	Independent events that regenerated	Transformation efficiency (%)
*LW1*	534	24	4.49	428	31	7.24
*NRT1.1*	500	13	2.6	-	-	-
Total	1034	37	3.57	428	31	7.24

PCR amplification of genomic DNA isolated from putative *lw1* mutant tissue followed by gel electrophoresis revealed successful multiplex editing. Data from four events are presented as examples (Fig. 2). When using primers spanning the target sites for Lw-1-gRNA1, Lw1-gRNA2, and Lw1-gRNA3, control non-edited plants produced the expected 900-bp amplicon. ILP1 event 1 (ILP-E1) produced three amplicons of approximately 800, 650 and 500-bp in length. Despite being the smallest fragment, the staining intensity of the ~ 500-bp amplicon was greater (Fig. 2F), indicating it was the most frequent edit. The size of the ~ 500-bp amplicon is consistent with the expected loss of 387 bp between *Lw1*-gRNA2 and *Lw1*-gRNA3, whereas the ~ 800-bp amplicon is consistent with a primary mutation at Lw1-gRNA3; both these deletion mutations were confirmed by Sanger sequencing (Fig. 2G and H). We did not recover sequence from the middle 650-bp fragment. Sanger sequencing of PCR amplicons that are the same size as the untransformed control identified additional mutations at each of the *Lw1* gRNA target sites (Fig. 2G-I). Two separate plants regenerated from ILP1 event 2 (ILP-E2) contained the same mutations. One mutation appears to result from double-strand breaks generated by Lw1-gRNA1 and Lw1-gRNA2, repaired by re-insertion of the cleavage fragment to generate a tandem duplication (Fig. 2G). Apparent point mutations are also observed near the Lw1-gRNA2 and Lw1-gRNA3 target sites. The ILP1-E3 event harbored single-base insertions at each of Lw1-gRNA1, Lw1-gRNA2 and Lw1-gRNA3. For one event in the H99 background (H99-E1), an amplicon of ~ 860 bp was produced because of a 40-bp deletion generated after cleavage and end-joining at the *LW1*-gRNA1 and *LW1*-gRNA2 sites (Fig. 2F and G). PCR amplification of these same events with primers spanning the Lw1-gRNA4 target sequence did not reveal any obvious differences in size compared to the 412-bp amplicon produced from non-edited plants. However, Sanger sequencing revealed small deletions in each of the ILP-E1, ILP-E2, and H99-E1 events. To summarize, Csy4 multiplexed CRISPR/Cas9 editing created multiple distinct mutations in all four gRNA target sites in the *LW1* gene.

### Mutagenesis of a gene family

**Fig. 3:**
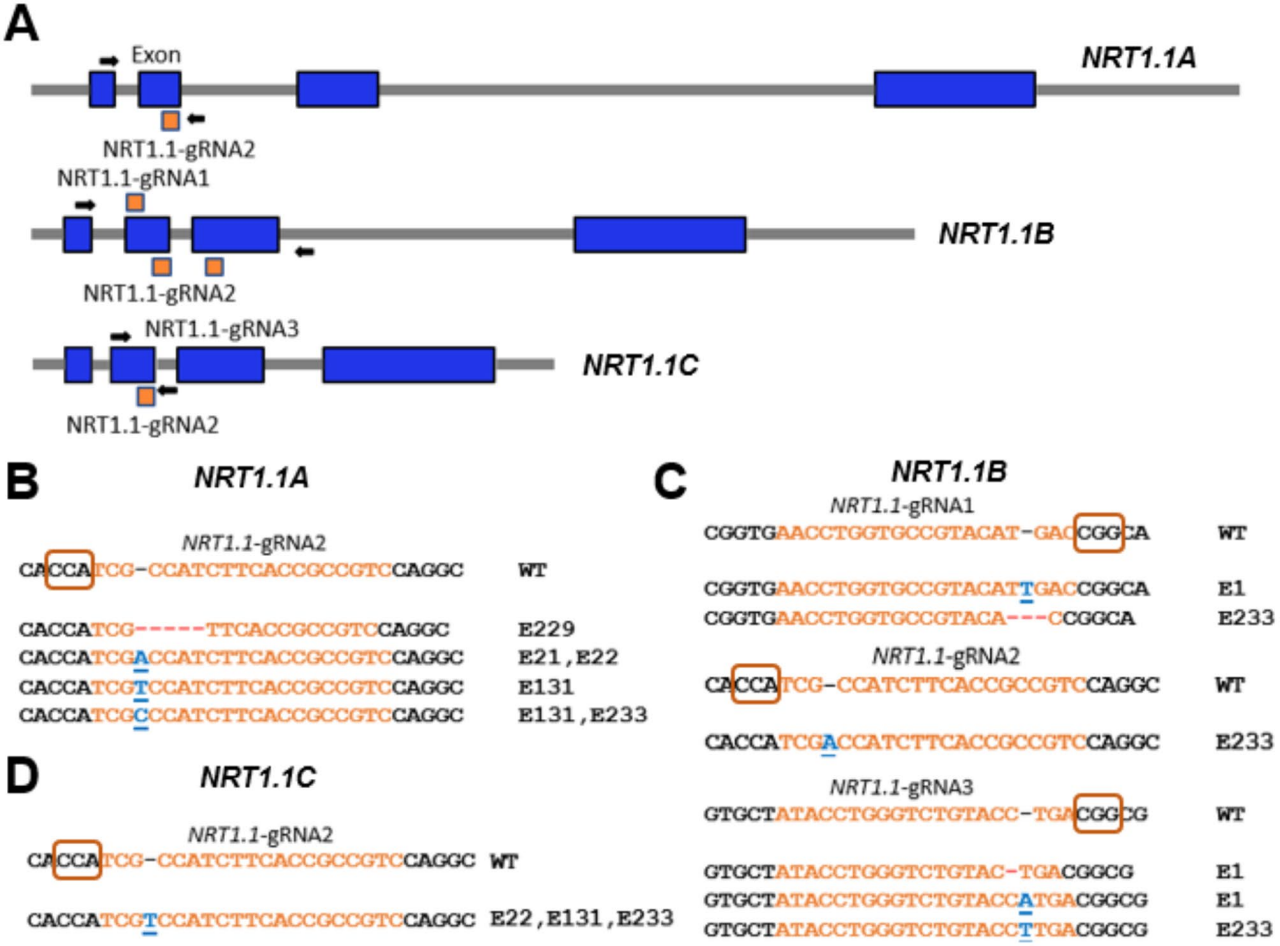
Mutagenesis of the maize *NRT1.1* gene family by multiplex CRISPR/Cas9 editing. **(A)***NRT1.1 A*, *B* and *C* gene models depicting exons (blue boxes), introns (grey lines) and the target sites for designed guide RNAs (orange boxes). The arrows on the gene models represent the gene-specific primers used for PCR confirmations of heritable edits in T1 plants. **(B**, ** C & D)** Sanger sequencing results of PCR products amplified using gene specific primers in gene-edited plants (guide RNA targets and PAM sequences are depicted in orange letters and boxes, respectively; red dashes represent deletions, bold blue underlined letters represent insertions)

The experiments with *LW1* demonstrated each of the construct elements was functional in multiplex genome editing. We next examined if we could use this method to edit multiple genes of a family using guide RNAs designed to target sequences conserved across all members, or unique to individual genes. Because of our interest in nitrogen utilization, we decided to edit the *NRT1.1* gene family of nitrate transporters (Fig. 3A). Maize contains four *NRT1.1* family members [33]. *NRT1.1 A* (Zm00001d024587) is located on chromosome 10, *NRT1.1B* (Zm00001d029932) and *NRT1.1 C* (Zm00001d029933) are tandemly-duplicated copies on chromosome 1, and *NRT1.1D* (Zm00001d027285) resides at the distal tip of the short arm of chromosome 1. Inspection of gene expression data for these genes at MaizeGDB.org shows that compared to *NRT1.1 A* and *NRT1.1B*, *NRT1.1 C* is weakly expressed and no expression is observed for *NRT1.1D*, which is likely a pseudogene. These expression differences were also observed for leaf tissue from multiple maize genotypes [9]. To simultaneously generate edits in each of the *NRT1.1 A*, *NRT1.1B* and *NRT1.1 C* genes, we designed three gRNAs: *NRT1.1-*gRNA2 targeted a perfectly conserved sequence in each gene, whereas *NRT1.1-*gRNA1 specifically targeted *NRT1.1B* and *NRT1.1-*gRNA2 specifically targeted *NRT1.1 C* (Fig. 3A). To design *NRT1.1-*gRNA2, the off-target requirement was relaxed to allow perfect matching at the three genes. All guide RNA targets are located within or upstream of several transmembrane domains within NRT1.1 proteins that are core for small molecule transport. The functionality of the *NRT1.1-*gRNAs at each of the three *NRT1.1* gene targets was verified using both in vitro Cas9 RNP assays (data not shown) and after transformation of maize leaf protoplasts (Supplementary Fig. 2).

Immature H99 maize embryos were bombarded with the linearized *NRT1.1* multiplex editing construct and thirteen resistant events were recovered, resulting in a transformation efficiency of 2.6% (Table 1). Of these 13 events, seven were confirmed to contain a mutation at one or more *NRT1.1-*gRNA target sites. All seven events were successfully regenerated into fertile T0 plants. Table 2 summarizes the spectrum of mutations obtained in the *NRT1.1* gene family that were identified by Sanger sequencing of PCR amplicons from the seven independent callus lines and three regenerated T0 plants from each event (Fig. 2B, C and D; Table 2). Overall, 19 different mutations were recovered from the three *NRT1.1* genes (Table 2). Among these mutations, 12 were an insertion of one or two base pairs and two others were small deletions of 1–18 base pairs. T0 edited plants ranged from chimeric, heterozygous, or homozygous for bi-allelic mutations.

**Table 2:** CRISPR/Cas9 editing outcomes for maize *NRT1.1* family genes

gRNA target T0 callus event	NRT1.1B1 mutant^1^, %^2^	NRT1.1B2 mutant^1^, %^2^	NRT1.1B3 mutant^1^, %^2^	NRT1.1A2 mutant^1^, %^2^	NRT1.1C2 mutant^1^, %^2^
1	WT	**WT** ^**3**^	WT	+1 (C), 46.7	-18, 51.1
1	WT	WT	WT	WT	+2, 45.3
21	WT	WT	WT	**+1 (A)**,** 46.5**	+1 (C), 54.8
22	**CE**	WT	WT	+1 (A), 50	+1 (A), 50
22	WT	WT	WT	+1 (T), 50	**+1 (T)**,** 50**
70	WT	WT	WT	WT	WT
131	WT	**CE**	WT	**+2**,** 16.2**	**+1 (T)**,** 21.9**
171	WT	WT	WT	+1 (A), 46.7	+1 (T), 8.4
229	WT	WT	WT	**-5**,** 45.1**	WT
233	**-3**,** 100**	**+1 (A)**,** 100**	**+1 (T)**,** 100**	**+1 (C)**,** 100**	**+1 (T)**,** 100**

^1^ Sequence change, WT = wild-type sequence, CE = chimeric edit

^2^ Proportion of edited DNA estimated by TIDE analysis

**Bold font** indicates mutation was also detected in Sanger sequencing of PCR amplicons from at least two of three regenerated T0 plants

^3^TIDE analysis of callus did not detect edit, but a + 1 (A) mutation was detected in two of three regenerated T0 plants

Eight different *nrt1.1a* mutations were generated, with a single ‘C’ insertion occurring in two events (E1 and E233) and a single ‘A’ insertion in both events 21 and 171 (Table 2). Three events (E22, E131 and E233) showed evidence of editing at *NRT1.1B*, but we were not able to precisely identify the nucleotide differences (Table 2). This result could indicate higher editing activity by the three gRNAs targeting *NRT1.1B* compared to a single gRNA targeting either *NRT1.1 A* or *NRT1.1 C*. Eight different mutations were observed in *NRT1.1 C*, including a single ‘T’ insertion found in four events (E22, E131, E171, and E233; Table 2). Among the 19 mutations observed in calli, 9 were also observed in DNA from seedling leaves of the regenerated T0 plants, at least three mutations for each of *NRT1.1 A*, *NRT1.1B* and *NRT1.1 C* (Table 2; Fig. 3B, C and D). Conversely, two new single base insertions were identified in T0 plants for *NRT1.1 A* (E131; Fig. 3B) and another in *NRT1.1B* near *NRT1.1-*gRNA3 (E1; Fig. 3C) that were not identified at the callus stage (Table 2). At the T0 plant stage, the unknown chimeric edits in *NRT1.1B* for the callus event 233 was resolved to a homozygous mutation comprised of a 3-bp deletion near *NRT1.1-*gRNA1, a single ‘A’ insertion near *NRT1.1-*gRNA2 and a single ‘T’ insertion at *NRT1.1-*gRNA3 (Table 2; Fig. 3C). The single base insertions in Event 21 (for *NRT1.1 A*) and Event 131 (for *NRT1.1 C*), as well as the 5-bp deletion for *NRT1.1 A* found in event 229, each showed approximately 50% editing in both callus and T0 plants, indicative of fully heterozygous edits (Table 2; Fig. 3B, C and D). Importantly, among the eight total events analyzed, three (22, 131 and 233) showed evidence of editing at each of the three targeted genes at the callus stage. Event 233 was exceptional in that bi-allelic edits were observed at all three possible guide RNA target sites in callus and all 3 T0 plants, indicating Csy4 based multiplex editing of the *NRT1.1* gene family could proceed to completion in a single transformation event (Fig. 3B, C and D). We cannot discount the possibility that this result could reflect a large deletion allele that fails to be amplified by the primers used; however, if such a deletion was present, it was apparently not transmitted to progeny.

The heritability of the *NRT1.1* gene family edits was assessed in T1 progeny produced from either self-pollination of T0 plants or crossing as either male or females with control H99 plants. The inheritance of edited alleles in T1 progeny was confirmed by Sanger sequencing of the PCR amplicons using primers flanking identified mutations in T0 plants from events E1 and E233 (Fig. 3B-D). All together, these results demonstrate that multiplexed Csy4 CRISPR/Cas9 editing system successfully created mutations in multiple genes of a multi-gene family.

### Genome sequencing reveals rare off-target mutations in maize genome

To identify potential off-target mutations generated by the *NRT1.1* genome editing system, we performed Illumina deep sequencing on T0 seedling leaf tissue from event 1. This sequencing run produced 70 million total paired reads which gives an average read depth of 8.75X across the maize genome. These sequence reads were aligned to Oh43, which is the most closely related inbred to H99 with a complete genome assembly [21]. Each of the 20 genomic regions predicted as off target sites for the three *NRT1.1-*gRNAs were visually inspected for sequence variation indicative of editing. The average read depth within these off-target regions was 13.6. When allowing up to three sequence mismatches, only one of these 20 potential regions showed evidence of off-target mutagenesis, which coincided with *NRT1.1D*, the 4th member of the *NRT1.1* gene family (Figure S3A). This putative edit contained a one base-pair mismatch 17 nucleotides from the PAM proximal region of the *NRT1.1-*gRNA2 target sequence (Figure S3B). The read depth at this site was 12 and 4 of these reads showed an A nucleotide insertion, indicative of a chimeric edit. In conclusion, the deep sequencing indicated that like prior genome editing experiments with maize, off target mutagenesis is not common, and only observed here at the locus already known to share the strongest match with intended editing targets.

### Design and validation of indel-selective PCR assays for efficient genotyping of edits

Although mutagenesis by multiplex genome editing is effective, the spectrum of mutations generated can present challenges in screening for initial edits or downstream analyses to characterize functions of either individual or desired combinations of mutations. Large (> 10-bp) deletion mutations can be easily screened by changes in PCR amplicon size, but small, often single-base indels that are the most common editing outcome are not detected by this approach. In vitro digestion assays with Cas9 ribonucleoproteins can reveal any type of mutation at a target site, but not the specific sequence changes, and are not amenable to high-throughput screening. Thus, we sought to design a general PCR-based approach to identifying and tracking small indel mutations.

**Fig. 4:**
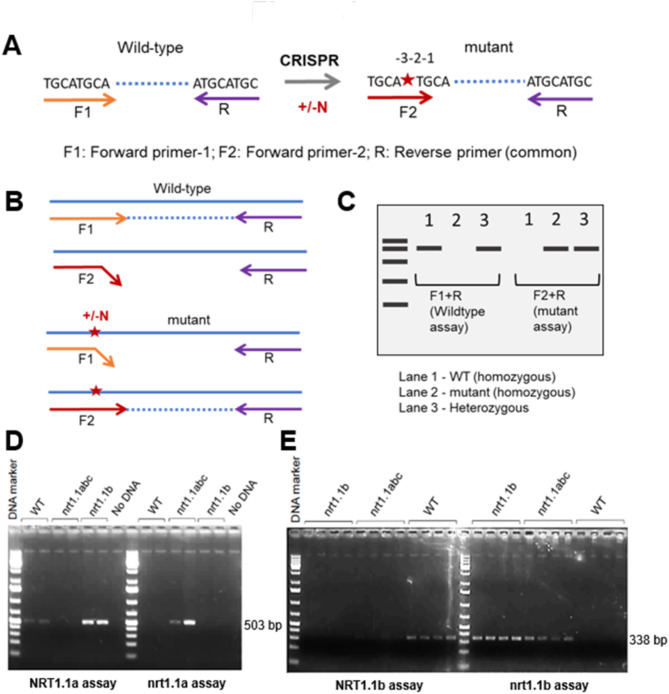
Indel-Selective PCR Assay. **(A)** Single base insertion/deletion (+/-N) created by CRISPR mutagenesis. To design wild type and indel allele specific forward primers (denoted by F1 & F2, respectively), the single base indel is positioned three nucleotides away (shown as -3) from the 3’ end of the primers. Reverse primer is common to assays for both wild-type and mutant alleles. **(B)** Illustration of 3’ end of allele-specific primer annealing only to their respective DNA template resulting in PCR amplification. **(C)** Schematic of the agarose gel electrophoresis analysis for wild type and mutant assay revealing the zygosity and genotype of tested DNA samples. **(D)** Gel image displaying PCR amplification of 503 bp DNA product for *NRT1.1a* and *nrt1.1a* mutant genotyping assays. **(E)**, Gel image displaying PCR amplification of 338 bp DNA product for *NRT1.1b* and *nrt1.1b* mutant genotyping assays

To design the indel-selective assay, we leveraged the observation that single nucleotide insertions or deletions created by CRISPR-Cas9 can also disrupt base-pairing of PCR primers at adjacent nucleotides. We reasoned that three nucleotide mismatches at the 3’end of the primer would likely disable primer extension by DNA polymerase, even when the remainder of the primer sequence anneals. PCR primers can thus be designed where the anticipated indel mutation(s) and the two adjacent nucleotides will have three mismatches with the original genomic target but will perfectly match mutated DNA (Fig. 4A and B). During PCR with a common reverse primer that matches both edited and reference alleles, the 3-nucleotide mismatch at the end of forward primers only allows proper primer annealing/extension to either the original or mutant sequence, therefore selectively amplifying one allele (Fig. 4B, C). If the extension of the next two nucleotides on one DNA strand does not enable design of forward primers that are indel-selective, for example due to repeated nucleotides, the assay can be designed to operate in the reverse orientation with indel-selective reverse primers and a common forward primer. Notably, successful PCR amplification from the same DNA sample with both assays (original sequence & mutant) reveals heterozygous genotypes (Fig. 4C, Supplementary Fig. 4).

We tested the indel-selective PCR approach to identify edited *nrt1.1* alleles. We used two events from the multiplex editing experiment, Event 233 that contains mutations in each of the three targeted *NRT1.1* genes (referred hereafter as *NRT1.1-abc*), and Event 1 that contains a single-base T insertion only in *NRT1.1B* (*nrt1.1b*). Assays using a forward primer matching the *NRT1.1a* reference allele amplified a DNA product of desired size (503 bp) from the wild-type control and *nrt1.1b* genomic DNA templates, but *nrt1.1abc* genomic DNA samples did not show any PCR amplification (Fig. 4D). Conversely, assays with a forward primer matching the *nrt1.1a* mutation with a single base C insertion amplified a DNA product of expected size only with *nrt1.1abc* genomic DNA template. A similar assay was designed to selectively amplify either the *NRT1.1B* reference sequence or the *nrt1.1b* mutant alleles in both events, which share a T insertion at the same position. For *NRT1.1b* alleles, the indel-selective PCR assay only worked in the opposite orientation i.e. common forward and indel selective reverse primers. The primer designed for the *NRT1.1B* reference allele selectively produced a ~ 338 bp DNA fragment only with wild type genomic DNA samples (Fig. 4E). The *nrt1.1b* mutant assay amplified the expected size DNA fragments in both the *nrt1.1abc* and *nrt1.1b* genomic DNA samples but not with wild-type DNA (Fig. 4E). Together, these observations demonstrate the indel-selective assays can identify edited mutations.

The above assays were subsequently employed to screen large progeny populations from events with multiplex edits, to identify individuals with single mutations, or desirable combinations of mutant alleles. During 2023 over both a summer field and fall greenhouse breeding cycles, 1152 plants were genotyped for *NRT1.1a* and/or *NRT1.1b* alleles (supplementary Figure S4). These plants derived from 46 families where the edited mutations are either being introgressed into additional genetic backgrounds or combined with other mutations in the common H99 background. Eight families were derived from crosses of the H99: *nrt1.1abc* triple mutant with H99, which subsequently segregated for individuals homozygous for either single *nrt1.1a* or tandemly-linked *nrt1.1b* and *nrt1.1c* mutations. From this genotyping campaign, we validated by Sanger sequencing five individuals identified as homozygous for either wild type or *nrt1.1* mutant alleles by our indel-selective PCR assays.

## Discussion

CRISPR/Cas9 genome editing technology has provided a significant boost towards crop improvement [48, 50]. Multiplexed genome editing using CRISPR/Cas9 has been achieved in maize but has been applied primarily to target two sites within the same gene. A number of studies have successfully mutagenized multiple genes [8, 10, 22, 25–27] but each have employed *Agrobacterium* transformation with constructs where gRNAs are expressed individually from RNA pol III promoters. The pioneering study by Qi et al. [35] successfully generated edits in multiple genes by expressing gRNAs separated by tRNA spacers from the same transcript, which were subsequently processed by the endogenous tRNA biogenesis pathway. However, gRNA expression was driven by RNA pol III promoters and relatively large constructs were delivered by *Agrobacterium* to Hi-II embryogenic callus. We present here a streamlined approach for multiplexed-genome editing of maize inbred lines by biolistics delivery of a compact single DNA fragment containing all editing components to Type I embryogenic callus, combined with indel-selective PCR to efficiently identify edited mutations.

Although multiplex editing has been achieved in other systems using the efficient vector design based on Csy4 processing [7], this report is the first to demonstrate its use in maize. The Csy4 multiplexing system offers two major advantages compared to RNA pol III promoters driving individual gRNAs. Firstly, Csy4 processing enables use of RNA pol II promoters that offer more flexibility and control in expression of editing components. Secondly, equal coexpression of gRNAs increases the probability each gRNA will be present in sufficient abundance to program Cas9 editing [30]. The compact nature of this vector design likely facilitates delivery of a greater proportion of intact DNA fragments by biolistics, where fragment size could be further reduced by substituting Cas12fs or other nucleases for the larger Cas9 protein. Similar to prior multiplex editing experiments in maize, the majority of T0 events generated (9 of 13 for *NRT1.1*) harbored at least one edited mutation, and the majority of these mutations (10 of 19 for the *NRT1.1* experiment, Table 2) were heritable. From the perspective of individual genes, editing frequency was on the order of 1 mutation per 100 initial embryos, or 1 heritable mutation from three to five transgenic events. The high frequencies of heritable edits from a few events per experiment compares similarly to other reports of multiplex editing in maize, where the majority of transgenic events produce heritable edits [8, 10, 23, 25, 27]. Similar to Feng et al. [14], we could not directly address the frequency of heritable edits from the *Lw1* experiments due to death or sterility of events, but a high frequency of somatic editing was observed (Fig. 2).

Although multiplex editing in maize has been achieved through biolistics delivery of editing components as separate fragments, this approach has so far been limited to targeting two sites within the same gene [15]. Transformation of 12 different inbreds with four DNA fragments (Cas9, two gRNAs, *nptII* selectable marker gene) was enhanced by co-bombardment with two additional constructs (six fragments total) encoding morphogenic regulators, which may have reduced mutation transmission rates. In contrast, our methods use one DNA fragment of ~ 12-kb that programmed efficient simultaneous editing of three paralagous *NRT1.1* genes, including recovery of a triple mutant line where all three genes were edited (*nrt1.1abc*). As has been observed in other maize editing experiments, many events are fully edited at the callus or T0 plant stage, indicating mutations were generated early in the transformation experiment. Although the lower frequency of quality single-copy events with biolistics delivery is certainly an issue in creating stable transgenic lines, a higher copy number of Cas9 and gRNA transgenes may benefit editing efficiency. Despite the potential for complex transgene insertions, most events generated with biolistics harbor transgene integration at a single locus [16, 44], and as shown here for *NRT1.1* edits, can be readily removed in subsequent generations via crossing. Furthermore, the ability to deliver significantly more DNA molecules to single cells via biolistics compared to *Agrobacterium* may increase the potential for mutagenesis from transient expression of editing components.

After establishing a successful Csy4 multiplex genome editing pipeline for the H99 inbred known to be competent for transformation, we also succeeded in creating transgenic events and edited mutations for the ILP1 inbred line that has never been transformed previously. Transformation efficiencies for the *LW1* and *NRT1.1* editing experiments in H99 were in line with previous work at about 3% [5]. Transformation efficiency for the *LW1* editing experiment in ILP1 was slightly higher at about 7%, which we have subsequently observed can be further increased with media optimization (data not shown). The same methods have succeeded in inducing Type I embryogenic callus and fertile regenerated plants for a wide diversity of maize genotypes [11, 20, 44]. More recently biolistic delivery to Type I calli of B104 produced transgenic events at 8% frequency [36]. The slower growth of Type I callus is a significant disadvantage with *Agrobacterium*-mediated transformation, where the rare transformed cells are less competitive in outgrowing *Agrobacterium* after co-cultivation, whereas this is not an issue with biolistics delivery. The methods described here open the possibility for direct editing of genotypes with other desirable traits, such as the unique kernel composition and nitrogen utilization phenotypes of ILP1 [42], thereby expanding the utility of CRISPR/Cas9 editing for maize functional genomics research.

The efficient mutagenesis of multiple targets achieved by multiplex editing complicates subsequent efforts to identify individual edits and track their inheritance. The *NRT1.1* gene editing experiment produced events with mutations in one gene, but the majority harbored mutations in more than one *NRT1.1* gene at a shared target sequence (e.g. *nrt1.1abc*), further complicating resolution of mutations in individual genes. As has been reported previously in Cas9-mediated editing in plants [3], we observed single-base insertions as the most common type of edited mutation (Fig. 4D). We demonstrate here that a single mismatched primer can selectively assay multiple classes of possible single-base indels. A few primers can be designed in advance to cover all possible anticipated single-base indels for a given target. Once the specific sequence change is determined, the indel-selective PCR assays can also be used for high-throughput genotyping of mutant populations. However, reliance on small mismatched primers can lead to variable impacts on amplification efficiency depending on local sequence context, the DNA polymerase, or PCR cycling conditions. Like many other PCR-based approaches to genotyping small indels, such as differential digestion by a restriction enzyme site spanning the edit or cleavage by in vitro Cas9-sgRNA assays, our indel-selective PCR strategy may not succeed in all cases.

Nevertheless, the indel-selective PCR assay does provide another option for the design of simple low-cost PCR assays to detect single-base edited alleles with a clear path for commercialization in the US and other countries that have declared these mutations as exempt from regulatory oversight [39]. Depending on the country, methods using biolistics delivery without any extraneous or vector backbone DNA may also be favored over *Agrobacterium* for gaining eventual regulatory approvals. The genome-editing methods reported here are also not encumbered by intellectual property beyond patents active for CRISPR/Cas9 mutagenesis, so could be used by the global maize research community. Considering the entire pipeline from design and assembly of vectors, DNA delivery, transformation and recovery of events, and genotyping for mutant identification, our approach is easier to implement for scientists new to genome editing. Each of the experiments reported here were conducted primarily by graduate and undergraduate students, where edits were confirmed within six months of initial gRNA design and starting with only 500 immature embryos, and the subsequent tissue culture readily handled by a single scientist. The Csy4 multiplex vectors and indel-selective PCR assays can also be used for genome editing of related grasses. We have already demonstrated multiplex editing of the LW1 genes from polyploid *Miscanthus* [41] and our group has recently applied this approach to generation and screening for single-base edits for sorghum genes using similar transformation methods [4]. We anticipate the versatility of the methods described here will enhance the utility of genome editing for functional genomics research in maize and other grasses.

## Materials and methods

### Guide RNA design and CRISPR/Cas9 expression vector construction

All guide RNAs (gRNAs) were designed using the CHOPCHOP web tool (https://chopchop.cbu.uib.no/). Default settings were used along with the B73 v4 genome for gRNA generation, efficiency scoring and off target prediction. All gRNAs chosen contained a GC content of 30–70%, a self-complementarity score of zero and no off-target sites with less than two mismatches. The off-target requirement was relaxed for *NRT1.1*-gRNA2 to allow for perfect matching at *NRT1.1 A*, *NRT1.1B* and *NRT1.1 C*. gRNAs designed from B73 sequence were confirmed to perfectly match target gene sequences present in the H99 and ILP1 genomes, as ascertained by perfect alignment of seedling RNASeq data from H99 [17] or Illumina genome shotgun sequence from ILP1 (accession SRP229948 at NCBI Short Read Archive) with the B73 v4 reference assembly.

The constructs for multiplex CRISPR/Cas9 experiments were generated using the base plasmids and Golden-gate cloning system [7] using appropriate primers (Supplementary Table 2). The specific plasmid components used were a monocot optimized Cas9 and Csy4 protein expressed under the maize Ubiquitin1 promoter and separated by a P2A self-cleaving peptide sequence (Addgene ID: 91036), a multiplexed gRNA cassette expressed with a Cestrum Yellow Leaf Curling Virus (CmYLCV) promoter and separated by Csy4 cleavage sites (Addgene ID: 91061), a blank homologous recombination module (Addgene ID: 91081) and a plasmid backbone containing the *NPTII* resistance gene driven by the 2 × 35 S promoter (Addgene ID: 91203).

### In vitro digestion of gene targets with Cas9 ribonucleoproteins

In vitro Cas9 Ribonucleoprotein (RNP) digestion was performed as previously described with minor changes [24]. Cas9-6X HIS protein was isolated following expression in *E. coli* using a Ni-NTA purification. Oligonucleotides for each designed gRNA were annealed, phosphorylated with T4 Polynucleotide Kinase (NEB Catalog #M0201S) and cloned into pT7-gRNA (Addgene Plasmid #46759). Synthetic RNA molecules of each gRNA target sequence and scaffold were created following NEB’s T7 RNA Synthesis Kit (Catalog #E2040S). Purified PCR products encompassing all gRNA target sites were amplified and eluted into RNase free water. The Cas9-gRNA digestion reaction was set up using Cas9 (1 µg), gRNA (375 ng), PCR product (200 ng), and 10X Cas9 reaction buffer (20mM HEPES, pH 7.5, 150 mM KCl, 10mM MgCl2, 0.5mM DTT). The reaction was incubated at 37 °C for 1 h for Cas9 digestion and 25 °C for 15 min after addition of proteinase K and at 56 °C for 10 min. Digested products were run on a 2% agarose gel at 100 V for 90 min and differences in band intensity were determined using ImageJ to calculate editing efficiencies.

### Plant materials, embryo excision and callus induction

All transformations in this study were conducted in maize inbred H99 and Illinois Low Protein (ILP1) lines, using the methods described in Shiva Prakash et al. [38]. Plants were either grown in a greenhouse or field, where ears from H99 were harvested between 10 and 12 days after pollination (DAP) and from ILP1 between 12 and 14 DAP. Prior to embryo excision, ears were surface sterilized by first drenching them in 70% ethanol, then submerging them into 30% bleach solution for 15 min, followed by multiple washes with sterile deionized water. After the final wash, the top 1–2 mm of kernel crowns was carefully removed with a scalpel. Immature embryos ranging from 1.5 to 2.0 mm in length were then aseptically excised. The excised embryos were placed scutellar side up on N6E media supplemented with auxin, 3% sucrose, 100 mg/L myo-inositol, 2.27 g/L proline, 100 mg/L casein hydrolysate and 0.25% phytagel. For H99, the auxin source was 2,4 D (2 mg/L), whereas for ILP1 auxin was supplied as Dicamba (3.315 mg/L) because of the higher frequency of regeneration with that media reported by Duncan et al. [11]. The plates were then incubated in the dark at 25 °C.

### Maize transformation

Four-day old embryos or embryogenic calli were placed onto N6OSM medium four hours prior to bombardment. N6OSM media is N6 medium with the addition of 0.69 g/L of proline and concentrations of 3.64% sorbitol and 3.64% mannitol. DNA fragments containing only the genome editing and plant selectable marker components were excised from plasmid vectors followed by gel-purification. The embryos/embryogenic calli were bombarded with gel-purified linear DNA fragment following precipitation onto 0.6 mM gold particles using spermidine and CaCl_2_ [2]. After 16–24 h of resting, the bombarded embryos/calli were transferred onto N6S medium containing 300 mg/L of paromomycin, plates were incubated in the dark at 25 °C, and subcultured every two weeks until resistant calli could be identified.

After ~ 8 weeks of selection, the resistant calli were moved onto Regeneration media-I (R-I) containing 300 mg/L of paromomycin and 5 mg/L 6-Benzoaminopurine (6-BAP). After 3–4 days on this media, the calli were transferred onto R-I media containing 300 mg/L of paromomycin only and incubated in dark at 25 °C. After 2–3 weeks the resistant calli were transferred into a growth chamber at 28 °C with 16 h/8 h light/dark cycle. Calli pieces were initially exposed to a lower light intensity by covering them with three layers of paper towels, then acclimatized to higher light by removing one layer of paper towels every two to three days. Regenerating plantlets were transferred onto Regeneration media II (R-II) containing 150 mg/L paramomycin in plastic cups (Solo). When plantlet shoots and roots grew to sufficient size in the plastic cups, they were transferred to moist soilless mix and allowed to develop under the same growth chamber conditions to acclimatize plants to lower relative humidity. Eventually, plants at the V3-V4 growth stage were transplanted to soil in 2 L volume pots for T1 seed production in a greenhouse. Maize protoplast transformation was conducted as described in Feng et al., [14]

### Screening of putative Transgenic lines

Genomic DNA of paromomycin-resistant calli or leaf tissue from regenerated plants was extracted and primers specific to Cas9 and guide RNA sequences (Supplementary Table 2) were used to identify events containing CRISPR/Cas9 components through PCR. Subsequently, an ELISA test for Cas9 (Epigentek Cat #: P-4060-96) was performed, according to the manufacturer’s protocol, to identify events expressing the protein. Sanger sequencing of PCR amplicons spanning guide RNA target sites were used to identify CRISPR/Cas9 induced mutations. Heterozygous, chimeric or complex alleles were analyzed using sequence trace decomposition as described in Brinkman et al. [6].

### Off-target CRISPR/Cas9 analysis

To identify and quantify any off-target mutations created during the *NRT1.1* CRISPR/Cas9 experiment, genomic DNA was extracted from *NRT1.1-*E1 callus tissue for deep sequencing. Genomic library preparation was conducted using the Nextera DNA Flex Library Prep Kit (Illumina Cat #: 20018704) according to the manufacturer’s protocol and sequencing was performed on an Illumina NovaSeq6000. Potential off target CRISPR/Cas9 recognition sites were initially predicted from DNA BLAST hits containing 3 or fewer mismatches with the *NRT1.1* gRNAs within Oh43, the line most closely related to H99 for which a full genome sequence was available. Then, quality-filtered Illumina reads from the *NRT1.1-*E1 callus DNA were aligned to predicted off-targets, and alignments were manually inspected using Integrated Genomics Viewer for evidence of CRISPR/Cas9 mediated mutagenesis.

### Molecular characterization of edited lines and screening of progenies

For efficient genotyping of single-base indel edits, we designed Indel-Selective PCR assays. The sequences of the edited events obtained from Sanger sequencing of PCR amplicons were used to design allele-specific primers which enable specific PCR amplification of either edited or reference alleles. The indel-selective assay employs two forward primers, one matching the original genomic target sequence where the 3’end nucleotide is positioned 2-bp away from the edited site, and a second similar forward primer where the third nucleotide from the 3’ end matches the single-base indel site, but now has a 3-bp mismatch with wild type sequence. The two forward primers are combined in separate PCRs with a common reverse primer that perfectly matches both reference and mutant sequences located downstream of the target site, at a position that enables efficient amplification of a product that is easily visualized by agarose gel electrophoresis. Primers were designed to have 18–22 bp size and Tm in the range 52–60 °C. Different Tm in the range were checked, and a Tm of 58 °C was observed to work best. The same primer design concepts were applied in reverse orientation for *NRT1.1B* alleles because the forward orientation did not yield indel-selective primers. Indel-selective reverse primers with the common forward primer produced the expected amplicon sizes and was able to distinguish between WT and mutant alleles.

Seeds from crosses with edited mutations were sown either in potting soil and grown in the greenhouse, or in the field during the summer months. Four to six leaf samples were collected from each plant with a hand-held single hole paper punch into 96-well tubes, lyophyllized, and genomic DNA extracted with a modified CTAB method [34]. Genomic DNAs were assayed by PCR with indel-selective assays for both reference and mutant alleles. Taq 5X Master Mix (New England Biolabs, M0285L) was used for PCR with following cycling conditions: initial denaturation at 95^o^C for 30 s, 35 cycles of 95^o^C 30 s, 58^o^C primer annealing for 30 s, 68^o^C extension for 30 s, followed by a final extension at 68^o^C for 10 min. PCR products were visualized by agarose gel electrophoresis.

## Electronic supplementary material

Below is the link to the electronic supplementary material.

Supplementary Material 1

## Data Availability

Sequence reads from edited H99 calli have been deposited at NCBI Short Read Archive under the accession number PRJNA1171432.

## References

[1] Aesaert S, Impens L, Coussens G, Van Lerberge E, Vanderhaeghen R, Desmet L et al. (2022) Optimized transformation and gene editing of the B104 public maize inbred by improved tissue culture and use of morphogenic regulators. Front Plant Sci, 13. 10.3389/fpls.2022.883847 PMC9072829 35528934

[2] Aulinger IE, Peter SO, Schmid JE, Stamp P. Gametic embryos of maize as a target for biolistic transformation: comparison to immature zygotic embryos. Plant Cell Rep. 2003;21:585–91. 12789434 10.1007/s00299-002-0556-7

[3] Bortesi L, Zhu C, Zischewski J, Perez L, Bassié L, Nadi R, et al. Patterns of CRISPR/Cas9 activity in plants, animals and microbes. Plant Biotechnol J. 2016;14:2203–16. 27614091 10.1111/pbi.12634 PMC5103219

[4] Brant EJ, Baloglu MC, Parikh A, Altpeter F. CRISPR/Cas9 mediated targeted mutagenesis of LIGULELESS-1 in sorghum provides a rapidly scorable phenotype by altering leaf inclination angle. Biotechnol J. 2021;16:2100237. 10.1002/biot.202100237 34343415

[5] Brettschneider R, Becker D, Lörz H. Efficient transformation of scutellar tissue of immature maize embryos. Theor Appl Genet. 1997;94:737–48.

[6] Brinkman Eva Karina and, van Steensel B. Rapid quantitative evaluation of CRISPR genome editing by TIDE and TIDER. In: Luo Y, editor. CRISPR gene editing: methods and protocols. New York, NY: Springer New York; 2019. pp. 29–44. 10.1007/978-1-4939-9170-9_3 30912038

[7] Čermák T, Curtin SJ, Gil-Humanes J, Čegan R, Kono TJY, Konečná E, et al. A multipurpose toolkit to enable advanced genome engineering in plants. Plant Cell. 2017;29:1196–217. 28522548 10.1105/tpc.16.00922 PMC5502448

[8] Char SN, Neelakandan AK, Nahampun H, Frame B, Main M, Spalding MH, et al. An Agrobacterium-delivered CRISPR/Cas9 system for high-frequency targeted mutagenesis in maize. Plant Biotechnol J. 2017;15:257–68. 27510362 10.1111/pbi.12611 PMC5259581

[9] Cheng C-Y, Li Y, Varala K, Bubert J, Huang J, Kim GJ, et al. Evolutionarily informed machine learning enhances the power of predictive gene-to-phenotype relationships. Nat Commun. 2021;12:5627. 34561450 10.1038/s41467-021-25893-w PMC8463701

[10] Doll NM, Gilles LM, Gérentes M-F, Richard C, Just J, Fierlej Y, et al. Single and multiple gene knockouts by CRISPR–Cas9 in maize. Plant Cell Rep. 2019;38:487–501. 30684023 10.1007/s00299-019-02378-1

[11] Duncan DR, Williams ME, Zehr BE, Widholm JM. The production of callus capable of plant regeneration from immature embryos of numerous Zea Mays genotypes. Planta. 1985;165:322–32. 24241136 10.1007/BF00392228

[12] Erenstein O, Jaleta M, Sonder K, Mottaleb K, Prasanna BM. Global maize production, consumption and trade: trends and R&D implications. Food Secur. 2022;14:1295–319.

[13] Feng C, Su H, Bai H, Wang R, Liu Y, Guo X, et al. High-efficiency genome editing using a dmc1 promoter-controlled CRISPR/Cas9 system in maize. Plant Biotechnol J. 2018;16:1848–57. 29569825 10.1111/pbi.12920 PMC6181213

[14] Feng C, Yuan J, Wang R, Liu Y, Birchler JA, Han F. Efficient targeted genome modification in maize using CRISPR/Cas9 system. J Genet Genomics. 2016;43:37–43. 26842992 10.1016/j.jgg.2015.10.002

[15] Gao H, Gadlage MJ, Lafitte HR, Lenderts B, Yang M, Schroder M, et al. Superior field performance of waxy corn engineered using CRISPR–Cas9. Nat Biotechnol. 2020;38:579–81. 32152597 10.1038/s41587-020-0444-0

[16] Gordon-Kamm WJ, Spencer TM, Mangano ML, Adams TR, Daines RJ, Start WG, et al. Transformation of maize cells and regeneration of fertile Transgenic plants. Plant Cell. 1990;2:603–18. 12354967 10.1105/tpc.2.7.603 PMC159915

[17] Hansey CN, Vaillancourt B, Sekhon RS, de Leon N, Kaeppler SM, Buell CR. Maize (Zea Mays L.) genome diversity as revealed by RNA-Sequencing. PLoS ONE. 2012;7:e33071. 22438891 10.1371/journal.pone.0033071 PMC3306378

[18] Hassan MM, Zhang Y, Yuan G, De K, Chen J-G, Muchero W, et al. Construct design for CRISPR/Cas-based genome editing in plants. Trends Plant Sci. 2021;26:1133–52. 34340931 10.1016/j.tplants.2021.06.015

[19] He Y, Zhang T, Yang N, Xu M, Yan L, Wang L, et al. Self-cleaving ribozymes enable the production of guide RNAs from unlimited choices of promoters for CRISPR/Cas9 mediated genome editing. J Genet Genomics. 2017;44:469–72. 28958488 10.1016/j.jgg.2017.08.003 PMC5736383

[20] Hodges TK, Kamo KK, Imbrie CW, Becwar MR. Genotype specificity of somatic embryogenesis and regeneration in maize. Bio/Technology. 1986;4:219–23.

[21] Hufford MB, Seetharam AS, Woodhouse MR, Chougule KM, Ou S, Liu J, et al. De Novo assembly, annotation, and comparative analysis of 26 diverse maize genomes. Sci (1979). 2021;373:655–62. 10.1126/science.abg5289 PMC8733867 34353948

[22] Hurst JP, Sato S, Ferris T, Yobi A, Zhou Y, Angelovici R, et al. Editing the 19 kda alpha-zein gene family generates non-opaque2-based quality protein maize. Plant Biotechnol J. 2024;22:946–59. 37988568 10.1111/pbi.14237 PMC10955486

[23] Kang M, Lee K, Finley T, Chappell H, Veena V, Wang K. (2022) An improved Agrobacterium-Mediated transformation and Genome-Editing method for maize inbred B104 using a ternary vector system and immature embryos. Front Plant Sci, 13. 10.3389/fpls.2022.860971 PMC9114882 35599865

[24] Liang Z, Chen K, Li T, Zhang Y, Wang Y, Zhao Q, et al. Efficient DNA-free genome editing of bread wheat using CRISPR/Cas9 ribonucleoprotein complexes. Nat Commun. 2017;8:14261. 28098143 10.1038/ncomms14261 PMC5253684

[25] Liu H-J, Jian L, Xu J, Zhang Q, Zhang, Maolin, Jin M, et al. High-Throughput CRISPR/Cas9 mutagenesis streamlines trait gene identification in maize. Plant Cell. 2020;32:1397–413. 32102844 10.1105/tpc.19.00934 PMC7203946

[26] Liu X, Zhang S, Jiang Y, Yan T, Fang C, Hou Q et al. (2022) Use of CRISPR/Cas9-Based Gene Editing to Simultaneously Mutate Multiple Homologous Genes Required for Pollen Development and Male Fertility in Maize. Cells, 11. 10.3390/cells11030439 PMC8834288 35159251

[27] Lorenzo CD, Debray K, Herwegh D, Develtere W, Impens L, Schaumont D, et al. BREEDIT: a multiplex genome editing strategy to improve complex quantitative traits in maize. Plant Cell. 2023;35:218–38. 36066192 10.1093/plcell/koac243 PMC9806654

[28] Lowe K, La Rota M, Hoerster G, Hastings C, Wang N, Chamberlin M, et al. Rapid genotype independent Zea Mays L. (maize) transformation via direct somatic embryogenesis. Vitro Cell Dev Biology - Plant. 2018;54:240–52. 10.1007/s11627-018-9905-2 PMC5954046 29780216

[29] Lowe K, Wu E, Wang N, Hoerster G, Hastings C, Cho M-J, et al. Morphogenic regulators baby boom and Wuschel Improve monocot transformation. Plant Cell. 2016;28:1998–2015. 27600536 10.1105/tpc.16.00124 PMC5059793

[30] Luo Y, Na R, Nowak JS, Qiu Y, Lu QS, Yang C, et al. Development of a Csy4-processed guide RNA delivery system with soybean-infecting virus ALSV for genome editing. BMC Plant Biol. 2021;21:419. 34517842 10.1186/s12870-021-03138-8 PMC8436479

[31] McCarty NS, Graham AE, Studená L, Ledesma-Amaro R. Multiplexed CRISPR technologies for gene editing and transcriptional regulation. Nat Commun. 2020;11:1281. 32152313 10.1038/s41467-020-15053-x PMC7062760

[32] Moose SP, Dudley JW, Rocheford TR. Maize selection passes the century mark: a unique resource for 21st century genomics. Trends Plant Sci. 2004;9:358–64. 15231281 10.1016/j.tplants.2004.05.005

[33] Plett D, Toubia J, Garnett T, Tester M, Kaiser BN, Baumann U. Dichotomy in the NRT gene families of dicots and grass species. PLoS ONE. 2010;5:e15289. 21151904 10.1371/journal.pone.0015289 PMC2997785

[34] Porebski S, Bailey LG, Baum BR. Modification of a CTAB DNA extraction protocol for plants containing high polysaccharide and polyphenol components. Plant Mol Biol Rep. 1997;15:8–15.

[35] Qi W, Zhu T, Tian Z, Li C, Zhang W, Song R. High-efficiency CRISPR/Cas9 multiplex gene editing using the Glycine tRNA-processing system-based strategy in maize. BMC Biotechnol. 2016;16:58. 27515683 10.1186/s12896-016-0289-2 PMC4982333

[36] Raji JA, Frame B, Little D, Santoso TJ, Wang K. (2018). *Agrobacterium*-and biolistic-mediated transformation of maize B104 inbred. Maize: Methods Protocols, 15–40. 10.1007/978-1-4939-7315-6_2 28986902

[37] Shiose L, Moreira JdosR, Lira BS, Ponciano G, Gómez-Ocampo G, Wu RTA, et al. A tomato B-box protein regulates plant development and fruit quality through the interaction with PIF4, HY5, and RIN transcription factors. J Exp Bot. 2024;75:3368–87. 38492237 10.1093/jxb/erae119

[38] Shiva Prakash N, Prasad V, Chidambram TP, Cherian S, Jayaprakash TL, Dasgupta S, et al. Effect of promoter driving selectable marker on corn transformation. Transgenic Res. 2008;17:695–704. 17952623 10.1007/s11248-007-9149-0

[39] Tachikawa M, Matsuo M. (2023) Divergence and convergence in international regulatory policies regarding genome-edited food: how to find a middle ground. Front Plant Sci, 14. 10.3389/fpls.2023.1105426 PMC9923018 36794228

[40] Tang X, Zheng X, Qi Y, Zhang D, Cheng Y, Tang A, et al. A single transcript CRISPR-Cas9 system for efficient genome editing in plants. Mol Plant. 2016;9:1088–91. 27212389 10.1016/j.molp.2016.05.001

[41] Trieu A, Belaffif MB, Hirannaiah P, Manjunatha S, Wood R, Bathula Y, et al. Transformation and gene editing in the bioenergy grass miscanthus. Biotechnol Biofuels Bioprod. 2022;15:148. 36578060 10.1186/s13068-022-02241-8 PMC9798709

[42] Uribelarrea M, Moose SP, Below FE. Divergent selection for grain protein affects nitrogen use in maize hybrids. Field Crops Res. 2007;100:82–90.

[43] Varotto, Serena. "Current Status and future prospective of genome editing application in maize." In A roadmap for plant genome editing, pp. 165-182. Cham: Springer Nature Switzerland, 2023.

[44] Wan Y, Widholm JM, Lemaux PG. Type I callus as a bombardment target for generating fertile Transgenic maize (Zea Mays L). Planta. 1995;196:7–14.

[45] Wang B, Lin Z, Li, Xin, Zhao Y, Zhao B, Wu G, et al. Genome-wide selection and genetic improvement during modern maize breeding. Nat Genet. 2020;52:565–71. 32341525 10.1038/s41588-020-0616-3

[46] Wang N, Ryan L, Sardesai N, Wu E, Lenderts B, Lowe K, et al. Leaf transformation for efficient random integration and targeted genome modification in maize and sorghum. Nat Plants. 2023;9:255–70. 36759580 10.1038/s41477-022-01338-0 PMC9946824

[47] Wang Z, Wan L, Xin Q, Zhang X, Song Y, Wang P, et al. Optimizing glyphosate tolerance in rapeseed by CRISPR/Cas9-based geminiviral donor DNA replicon system with Csy4-based single-guide RNA processing. J Exp Bot. 2021;72:4796–808. 33872346 10.1093/jxb/erab167

[48] Zhang D, Zhang Z, Unver T, Zhang B. CRISPR/Cas: A powerful tool for gene function study and crop improvement. J Adv Res. 2021;29:207–21. 33842017 10.1016/j.jare.2020.10.003 PMC8020163

[49] Zhang J, Fengler KA, Van Hemert JL, Gupta R, Mongar N, Sun J, et al. Identification and characterization of a novel stay-green QTL that increases yield in maize. Plant Biotechnol J. 2019;17:2272–85. 31033139 10.1111/pbi.13139 PMC6835130

[50] Zhu H, Li C, Gao C. Applications of CRISPR–Cas in agriculture and plant biotechnology. Nat Rev Mol Cell Biol. 2020;21:661–77. 32973356 10.1038/s41580-020-00288-9

