# Supplementary material for: Efficient mutagenesis and genotyping of maize inbreds using biolistics, multiplex CRISPR/Cas9 editing, and Indel-Selective PCR
Source: Plant Methods. 2025 Mar 25;21:43. doi: 10.1186/s13007-025-01365-w (PMC11934539; doi:10.1186/s13007-025-01365-w)
Supplement: Supplementary file 1 — Supplementary Material 1 [file 13007_2025_1365_MOESM1_ESM.docx]

**Supplementary figures and tables:**


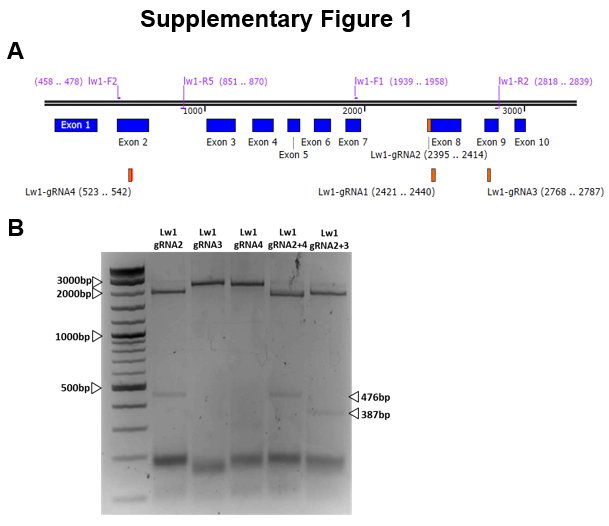


**Supplementary Figure 1:** **Demonstration of gRNA-targeted cleavage of *LW1* DNA by Cas9 RNP in vitro digestion assays.** **(A)** Schematic of *LW1* gene model with target sites for four gRNAs, and primers to amplify amplicons to test guide RNAs. **(B)** Gel electrophoresis of DNA fragments incubated with Cas9 RNPs loaded with either single or combinations of sgRNAs. Arrows indicate the sizes of fragments expected after cleavage. Guide RNAs 3 and 4 are close to primers to amplify amplicons and therefore did not produce detectable bands.


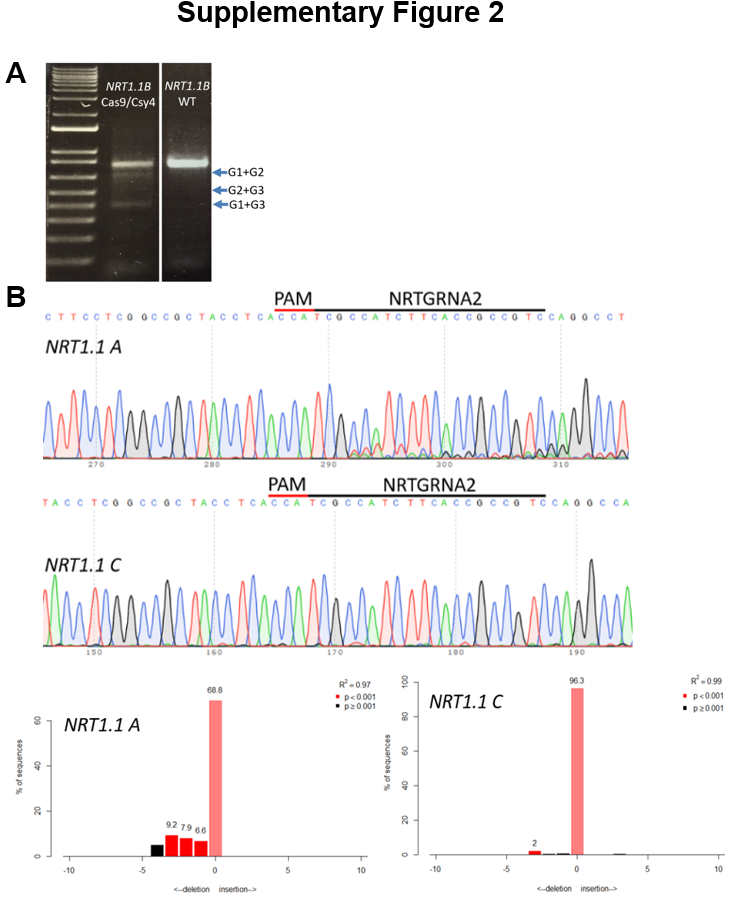


**Supplementary Figure 2:** Confirmation of NRT1.1-gRNA activity in maize leaf protoplasts. The *NRT1.1* editing construct was transfected into protoplasts using PEG, genomic DNA extracted from protoplasts after 48 hours, followed by PCR amplification of DNA spanning the NRT1.1-gRNA target sites. (**A**) Gel electrophoresis shows DNA fragment sizes consistent with deletions in the *NRT1.1B* gene resulting from multiplex editing (blue arrows). (**B**) Sanger sequencing chromatograms and predicted proportion of editing for PCR products spanning the NRT1.1-gRNA2 cleavage site from the *NRT1.1A* and *NRT1.1C* genes.


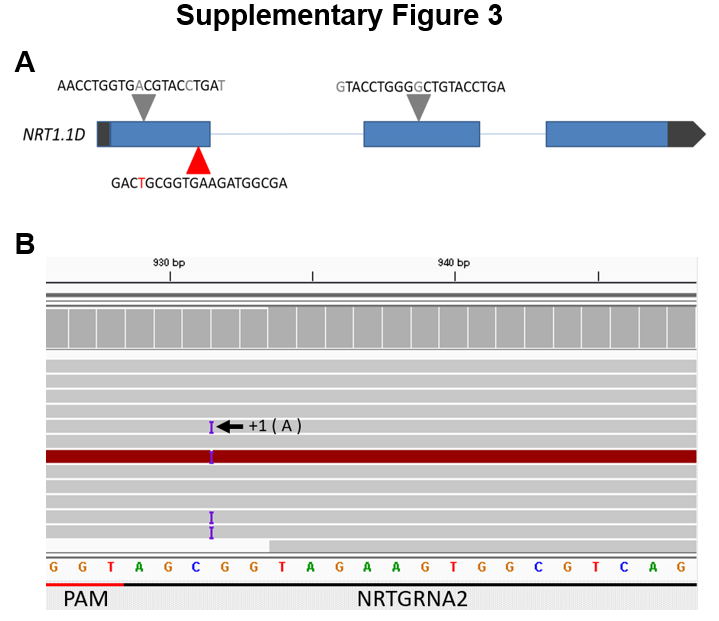


**Supplementary Figure 3:** Off target assessment of the CRISPR events. **(A)** Schematic representation of the off-target gene *NRT1.1D* gene model. The red triangle one base mismatch with the reference genome in the region complementary to guide RNA 2. **(B**) Integrated genome viewer (IGV) browser screenshot showing the insertion of nucleotide ‘A’ resulting in the off-target mutagenesis.

**
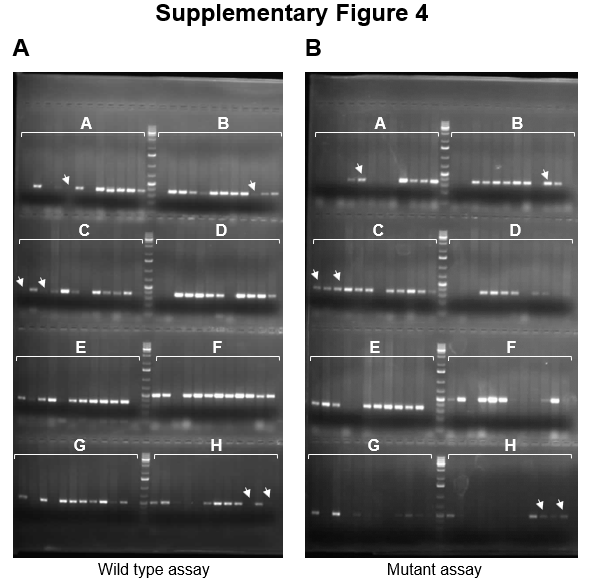
**

**Supplementary Figure 4: Representative gel picture of population segregating for *nrt1.1a* and *nrt1.1b*, to deconvolute homozygous mutant lines. (A)** Gel image depicting presence of *Nrt.1b* (rows A-E) and *Nrt1.1a* (rows F-H) wild type alleles in segregating maize lines. **(B)** Gel image displaying presence of nrt1.1b (rows A-E) and nrt1.1a (rows F-H) mutant alleles in segregating maize population. Arrow heads indicate the desired mutant lines showing bands for presence of mutant allele and absence of wild type allele.

**Supplementary Table 1: Alignment results for whole genome sequencing of DNA from callus event 233. From the NRT1.1 multiplex editing experiment.**

| **Off-target coordinates** | **Percent identity** | **Alignment length** | **Mismatches** | **Sequence** | **Edit** | **Notes** |
| --- | --- | --- | --- | --- | --- | --- |
| chr10:81131621-81131643 | 100 | 23 | 0 | CCATCGCCATCTTCACCGCCGTC | Y | *nrt1.1a* |
| chr1:95032630-95032652 | 100 | 23 | 0 | CCATCGCCATCTTCACCGCCGTC | N | *nrt1.1b* |
| chr1:95089208-95089186 | 100 | 23 | 0 | CCATCGCCATCTTCACCGCCGTC | Y | *nrt1.1c* |
| chr1:1773343-1773365 | 95.65 | 23 | 1 | CCATCGCCATCTTCACCGCAGTC | Y | *nrt1.1d* |
| chr10:30075995-30076017 | 91.3 | 23 | 2 | CCATCGCCATCGTCACCGTCGTC | N |  |
| chr8:61724083-61724061 | 91.3 | 23 | 2 | CCATCGCCATCTTCGCCGTCGTC | N |  |
| chr6:36488051-36488073 | 91.3 | 23 | 2 | CCATCGCCATCGTCATCGCCGTC | N |  |
| chr4:57413310-57413289 | 90.91 | 22 | 2 | CATCGCCATCTTCGCTGCCGTC | N |  |
| chr9:14264778-14264799 | 90.91 | 22 | 2 | CATGGCCATCATCACCGCCGTC | N |  |
| chr5:29734780-29734760 | 100 | 21 | 0 | CCATCGCCATCTTCACCGCCG | N |  |

**Supplementary Table 2: List of primers used in this study.**

| **For *LW1* editing experiment** | | |
| --- | --- | --- |
| **Primer Name** | **Primer Sequence (5' --> 3')** | **Purpose** |
| T7_lw1_g2_F | TAGGACCATATGTTTGATTCCTCA | gRNA cloning into T7 plasmid |
| T7_lw1_g2_R | AAACTGAGGAATCAAACATATGGT |  |
| T7_lw1_g3_F | TAGGGAAAAATAACTGGTTACCCG |  |
| T7_lw1_g3_R | AAACCGGGTAACCAGTTATTTTTC |  |
| T7_lw1_g4_F | TAGGGGAGGCCTACGGGTTCTGCT |  |
| T7_lw1_g4_R | AAACAGCAGAACCCGTAGGCCTCC |  |
| CSY_LW1GRNA1 | TCGTCTCCATAGCATCTTGTCTGCCTATACGGCAGTGAAC | gRNA cloning into CRISPR/Cas9 expression vector cassette |
| REP_LW1GRNA1 | TCGTCTCACTATGTATCAGCGTTTTAGAGCTAGAAATAGC |  |
| CSY_LW1GRNA2 | TCGTCTCCTTTGATTCCTCACTGCCTATACGGCAGTGAAC |  |
| REP_LW1GRNA2 | TCGTCTCACAAACATATGGTGTTTTAGAGCTAGAAATAGC |  |
| CSY_LW1GRNA3 | TCGTCTCCCAGTTATTTTTCCTGCCTATACGGCAGTGAAC |  |
| REP_LW1GRNA3 | TCGTCTCAACTGGTTACCCGGTTTTAGAGCTAGAAATAGC |  |
| CSY_LW1GRNA4 | TCGTCTCCCCGTAGGCCTCCCTGCCTATACGGCAGTGAAC |  |
| REP_LW1GRNA4 | TCGTCTCAACGGGTTCTGCTGTTTTAGAGCTAGAAATAGC |  |
| lw1-F1 | CACTTCATGGCCTTCAATAC | edit characterization |
| lw1-R2 | ACCTTATCTGGAGTTGAGGCAC |  |
| lw1-F2 | GTCATCAAGACGCTCAAGGAG |  |
| lw1-R5 | TGCAGTTAAGGCACGAACAC |  |
| **For *NRT1.1* editing experiment** | | |
| **Primer Name** | **Primer Sequence (5' --> 3')** | **Purpose** |
| CSY_NRTGRNA1 | TCGTCTCCTTCACCGCCGTCCTGCCTATACGGCAGTGAAC | gRNA cloning into CRISPR/Cas9 expression vector cassette |
| REP_NRTGRNA1 | TCGTCTCATGAAGATGGCGAGTTTTAGAGCTAGAAATAGC |  |
| CSY_NRTGRNA2 | TCGTCTCCCGGCACCAGGTTCTGCCTATACGGCAGTGAAC |  |
| REP_NRTGRNA2 | TCGTCTCAGCCGTACATGACGTTTTAGAGCTAGAAATAGC |  |
| CSY_NRTGRNA3 | TCGTCTCCAGACCCAGGTATCTGCCTATACGGCAGTGAAC |  |
| REP_NRTGRNA3 | TCGTCTCAGTCTGTACCTGAGTTTTAGAGCTAGAAATAGC |  |
| 086496_4F | GCCATGATCCTAGGTTGGTT | *NRT1.1A* edit characterization |
| 086496_4R | GTCGTCCCGAGTTTGTGG |  |
| 161459_4F | GGATACTGCGTCGGATGG | *NRT1.1B* edit characterization |
| 161459_9F | TGTGTGGTCCAGAGTCCTGA |  |
| 161459_5R | CGACACGCTGGACTTGAG |  |
| 161459_9R | CCCATCGATCTCTTTGAACTAC |  |
| 112154_2F | TTTGTGGTCTCGTGAAGGTG | *NRT1.1C* edit characterization |
| 112154_5R | AATTAGCCATCAGCGTGTCC |  |
| **For indel selective PCR genotyping** | | |
| **Primer name** | **Sequence** | **Purpose** |
| NRT1.1A_G2F | CCGCTACCTCACCATCGCCA | For *NRT1.1A* genotyping |
| nrt1.1a_e1_G2F | CCGCTACCTCACCATCGCCCA |  |
| NRT1.1A_I2R | CCACCGCACCACCGTGCAT |  |
| 161459 _4F | GGATACTGCGTCGGATGG | For *NRT1.1B* genotyping |
| NRT1.1b_3_G1R | GAGGTGCATGGTGCCGGTCATG |  |
| nrt1.1b_3_G1R | GAGGTGCATGGTGCCGGTCAAT |  |
